# Pertinence of contact duration as edge feature for epidemic spread analysis

**DOI:** 10.1038/s41598-025-94637-3

**Published:** 2025-03-28

**Authors:** Ramya D. Shetty, Shrutilipi Bhattacharjee

**Affiliations:** 1 Department of Information and Communication Technology, Manipal Institute of Technology, Manipal Academy of Higher Education, Manipal, 576104 India; 2 Department of Information Technology, National Institute of Technology Karnataka, Surathkal, 575025 India

**Keywords:** Engineering, Mathematics and computing

## Abstract

Identifying superspreading nodes has attracted greater attention because of its wide practical significance in various applications. Existing studies consider the edges mostly equally while designing the algorithms for the unweighted contact networks, where each connection explicitly shows whether the individuals are in contact or not. It will not consider other relevant information in the context of epidemiology study or infectious disease spread, such as proximity or total time spent between the contact nodes. The recent studies focused on the weighted network, where most of the methods have computed the edge weights by utilizing degree and k-shell measure, which captures the topological structure of the network but not the interaction duration between pair of contacts. In this study, we mainly aim to generate weighted networks to model the pathogen spread by optimal calculation of the edge weight in terms of contact duration (time spent) between individual contacts. Leveraging this interaction duration as the edge weight, we further design a novel technique, namely Real Weighted Influence (*RWInf*), for identifying the superspreading nodes during an epidemic outbreak. The empirical study revealed that the proposed approach outperforms with an improvement of 0.146–0.473 kendall’s score in comparison with baseline approaches.

## Introduction

Human social behavior plays an essential role in pathogen spread. The close proximity between contacts determines key insights about social interactions in human communities^1,2^. This type of social behavior is well captured with the advancement in technology and helps record human social connections^3^ etc. Hence, contact networks play a very important role in identifying superspreader in the case of infectious disease epidemiology. The study^4^ addresses a few challenges of effective use of contact networks for the epidemiology study and also highlights that the accurate assessment of the link strength between the individuals can make the network studies significantly more effective. Vanhems et al.^5^ show that wearable proximity sensors provide an optimal way of measuring contact paths between pairs of individuals. This data is also helpful in estimating potential infection transmission routes. The Génois et al.^6^ demonstrate that contact networks not only provide information about the interactions but also deal with the dynamics (temporal information) of interactions. This contact network also helps design effective vaccination strategies by identifying more dominant nodes.

Early superspreader identification algorithms assumed that all nodes interacted with one another at a constant rate and treated all edges as identical. The studies^7–9^, in identifying the influential node, where they modeled the graph as an unweighted graph, and the connection between nodes exhibit binary relation either 1 or 0, representing nodes are connected or disconnected, respectively. These studies utilize real-world networks such as facebook, email, music, transportation, etc. Identifying superspreaders mainly depends on the local structure, semi-local structure, global structure, and combination of both local and global structure of the network. The following are the studies related to superspreaders identification focusing on acquiring information from different levels of networks. Local centrality mainly focuses on the local information or the direct neighbor’s information of a node^10,11^. The study^12^ shows the effectiveness of employing semi-local measures based on redefining the topological distance with varying radius. Recently proposed algorithm supporting semi-local measure by Namtirtha et al.^13^, where the node ranking is done by counting the triangular connections of a node along with its neighboring nodes. Global centrality approach captures entire information about the network with the help of betweenness^14^, closeness^15^, k-shell decomposition^16^, hybrid rank approach^17^, eigenvector centrality^18^ etc. Our primary research^19^ recently studied the degree of heterogeneity in contact structure by leveraging local and global properties of the network in the context of influential node identification^8^. The above-highlighted studies solely model the interaction between individuals in unweighted networks. They will not consider important information about the edge features/attributes representing the probability of the information/infection propagation between pairs of nodes in case of disease spreading networks. To overcome such limitations, various studies extend the unweighted complex network analysis for the weighted network analysis^20–22^.

The studies on weighted networks signify the importance of edge features or connection strength to optimize complex network analysis more effectively. The Wei et al.^20^ proposed a weighting scheme, where edge weight is generated by adding the degree of the connecting nodes. The study^21^ makes use of the power-law function of degree measure to compute the edge quantity using tunable parameter $\alpha = 1$. The studies^22–24^, support weighted network analysis by utilizing degree, k-shell, and tunable parameters to yield good performance in the case of superspreader identification. These studies compute the connection strength or edge weight using either degree centrality or k-shell centrality measure. Mainly these centrality measures capture network topology, and they can be used as link strengths or edge weights to construct weighted networks.

In case of epidemiology studies, human contact networks are more often used^4,25,26^. Quantifying edge weight or link strength with data availability may be more effective for locating each of the virus spreaders in the network^4^. Danon et al.^27^ carried out study on contact networks showcasing the fundamental need for contact networks in epidemiology study and providing a unique way of estimating the significance of the different modes of pathogen transmission from contact tracing data. The study^4^ also addresses the challenges of using contact networks for epidemiology study on virus propagation relies on the proximity of contact or the length of interaction, i.e., duration or frequency of interaction between the infected and susceptible individuals. Modeling the dynamics of pathogen spread using contact networks is challenging however, it can be addressed by modeling individual interactions. In degree measure, it represents whether a connection with the neighbors is present or not. When we include edge weight, it not only indicates the existence of a connection but also quantifies the interaction (e.g., how much time individuals have spent together). This additional dimension offers more precise insights into how infections spread through interactions among individuals and provides a basis for approximating the transmission rate of the virus. This study also highlights that when we can compute the edge weight or strength robustly, we can analyze or explore crucial information about the network more effectively. With this insight from the above studies, we have attempted to derive the edge weight in terms of contact duration between individuals from the human contact networks to identify superspreading individuals effectively. To the best of our knowledge, this is the first approach to propose a method for computing edge weights using human contact networks and leveraging them to identify influential nodes^4,25,26^.

This work aims to model contagious disease spread by deriving appropriate edge features and utilizing these features on superspreading node identification in human contact networks. This area of research is suitable for the various applications related to epidemiology. The main contributions of the work are as follows:

- Proposing a novel algorithm to generate optimal edge weight in terms of duration of the interaction among the contacts in the network;
- Proposing a novel approach *RWInf* to leverage connection structure and derived edge features to locate superpsreading nodes in the human contact networks;
- Analyzing the difference between edge weights derived from the centrality measures and the real properties of the interaction duration between individuals;
- To validate the efficacy of the *RWInf* technique with eight different indexing methods on six different complex networks.

The rest of the sections are organized as follows: section “Baseline algorithms” details the comparison algorithms used in the study for comparison. Section “Problem statement” highlights the need for the study with the relevant use cases. Section “Proposed method” discusses the novel contribution followed by the experimental setups. “Results and discussion” section provides details of different experiments and presents the results of the empirical analysis. Finally, the “Conclusions” and future aspects are presented in the next section.

## Baseline algorithms

Real contact networks are weighted in practice, and their connection weights represent significant and precise characteristics of the underlying systems. Here, we discuss some baseline algorithms on weighted networks, which are used in this work for comparison with our work.

###  Weighted k-shell decomposition approach-1 ($W_{k-shell}$)

The $W_{k-shell}$^28^ approach takes into account both node’s degree and the edge weights to assign a weighted degree ($k'$) to each node. Finally, $k'_{v_i}$ is defined as follows:

1 $$\begin{aligned} k'_{v_i} = \left[ k_{v_i} ^ {\alpha _{1}} \left( \sum _{v_j} W_{{v_i}{v_j}} \right) ^ {\beta _{1}} \right] ^ \frac{1}{\alpha _{1} + \beta _{1}} \end{aligned}$$

where, $k_{v_i}$ denotes degree of the node ${v_i}$, $W_{{v_i}{v_j}}$ denotes link weight between pair of nodes ${v_i}$ and ${v_j}$, and the value of $\alpha _{1} = \beta _{1} = 1$ (as set in^28^).

### Weighted k-shell decomposition approach-2 (*Wks*)

In *Wks*^20^ approach, edge weight is computed using $W_{{v_i}{v_j}} = k_{v_i} + k_{v_j}$ where $k_{v_i}$ and $k_{v_j}$ represent the degree of the connected node pair $(v_i, v_j)$. Then assigning weighted degree ($k_{v_i}^W$) for each node is formulated as follows:

2 $$\begin{aligned} k_{v_i}^W = \alpha _1 * k_{v_i} + (1 - \alpha _{1}) \sum _{v_j \in \phi _{v_i}} W_{{v_i}{v_j}} \end{aligned}$$

where $\phi _{v_i}$ denotes the one hop direct neighbor set of $v_i$, $\alpha _{1}$ = 0.5 (as set in^20^).

### Weighted k-shell degree neighborhood method ($ksd^w$)

The $ksd^w$^13^ approach is the combination of the degree and the k-shell influence with the combination of tunable parameters $\alpha _{1}$ and $\mu _{1}$. Initially, the edge weight is computed as follows:

3 $$\begin{aligned} W_{{v_i}{v_j}} = (\alpha _{1} * k_{v_i} + \mu _{1} * ks_{v_i}) * (\alpha _{1} * k_{v_j} + \mu _{1} * ks_{v_j}) \end{aligned}$$

where $k_{v_i}$ and $k_{v_j}$ denote the degree of nodes $v_i$ and $v_j$, respectively, the tunable parameters vary from [0, 1], and their values are decided based on the giant component^29^. This is helpful to verify whether the network is fully connected hence, information/infection propagation can flow across all the nodes. Once we compute the edge weight, each of the node influences is calculated as follows:

4 $$\begin{aligned} ksd^W_{v_i} = \sum _{v_j \in \phi _{v_i}} W_{{v_i}{v_j}} \end{aligned}$$

where $\phi _{v_i}$ is the set of neighboring node of $v_i$, and $W_{{v_i}{v_j}}$ denotes the link weight of the connected nodes $v_i$ and $v_j$ respectively.

### Network Global Structure-based Centrality (*ngsc*)

The ngsc^22^ algorithm is utilized to identify the spreading nodes in the different types of complex networks connectivity structures, and its formulation shown in the below equation. Here, $\phi _{v_i}$ represents the set of direct connected nodes of $v_i$, $ks_{v_i}$ and $ks_{v_j}$ represents the k-shell values of $v_i$ and $v_j$, respectively. The $t_1$ and $t_2$ represent tunable parameters and its values are based on the network giant component.

5 $$\begin{aligned} \begin{aligned} ngsc_{v_i} = \sum _{v_j \in \phi _{v_i}} (t_1 * ks_{v_i} + t_2 * k_{v_i}) + (t_1 * ks_{v_j} + t_2 * k_{v_j}) \end{aligned} \end{aligned}$$

### *HIC* measure is the combination of H-index, k-shell measure, and clustering coefficient

HIC measure^30^ introduced an approach to measure the significance of the edge by incorporating different parameters, such as neighborhood information, position, and network connectivity structure. The edge weight $W_{v_iv_j}$ is computed as follows:

6 $$\begin{aligned} W_{{v_i}{v_j}} = \frac{H_{v_i} * I_{v_i}}{1 + C_{v_i}} + \frac{H_{v_j} * I_{v_j}}{1 + C_{v_j}} \end{aligned}$$

where the H-index and clustering coefficient of the connected pair of nodes $(v_i, v_j)$ are represented as ($H_{v_i}$, $H_{v_j}$) and ($C_{v_i}$, $C_{v_j}$), respectively. $I_{v_i}$ and $I_{v_j}$ represents k-shell iteration factor of the node $v_i$ and $v_j$. Once the potential edge weight is computed, then each of the network node influence will be the cumulative influence of its directly connected neighborhood, and it is defined as follows:

7 $$\begin{aligned} HIC_{v_i} = \sum _{v_j \in \phi _{v_i}} W_{{v_i}{v_j}} \end{aligned}$$

where $\phi _{v_i}$ denotes connected neighbor set of node $v_i$, and $W_{{v_i}{v_j}}$ represents weights on the edge between the node pair ($v_i, v_j$).

### Weighted degree centrality $C_{D}^W$

The weighted degree measure^31,32^ has been extended to the cumulative edge weights of the directly connected neighborhood and is given as follows:

8 $$\begin{aligned} C_{D_{v_i}}^W = \sum _{v_j} ^ V W_{{v_i}{v_j}} \end{aligned}$$

where $W_{{v_i}{v_j}}$ denotes the weighted adjacency matrix, in which the value $W_{{v_i}{v_j}} \ge 0$ if a contact exists between individuals, and the edge weight, quantifies the total time spent between individuals.

### Weighted betweenness centrality $C_{B} ^ W$

Betweenness centrality proposed by^33^ has been extended to generalize the centrality for weighted networks^31,34^. It is modeled as follows:

9 $$\begin{aligned} C_{B_{v_i}} ^ W = \sum _{v_j} \sum _{v_k} \frac{g_{v_j v_k} ^ W (v_i)}{g_{v_j v_k} ^ W} \end{aligned}$$

Depending on edge weight, the number of the shortest path between nodes $v_j$ and $v_k$ is computed and shown by the symbol $g_{v_j v_k} ^ W$, and the number of such paths passes through node $v_i$ is measured and represented as $g_{v_j v_k} ^ W (v_i)$.

### Weighted closeness centrality $C_{C}^W$^31,34,35^

Weighted Closeness centrality relies on the weights of the length of the paths from each node to all other nodes in the network and is measured as follows:

10 $$\begin{aligned} C_{C_{v_i}}^W = \left[ \sum _{v_j}^ V dist_{v_i v_j} ^ W \right] ^ {-1} \end{aligned}$$

where $dist_{v_i v_j}^W$ is the shortest route based on the edge weight among the nodes $v_i$ and $v_j$, and *V* represents the total number of nodes in the network.

## Problem statement

Most of the indexing methods proposed so far mainly focused on unweighted networks, where the edge signifies the nodes are connected or disconnected; in other words, the relationship between nodes is treated equally. Very few studies focus on the weighted networks^20–22,24,30^, where the edge weights are derived using degree or k-shell values, repeatedly captures topology structure of the network, but not represent the interaction duration between contacts. Accurately quantifying edge weight is essential because information/infection diffusion occurs via an edge or interaction among the individuals. Furthermore, the edge indicates how fast the infection propagation spreads or how quickly the information diffusion may occur in the network. Edge between pair of nodes has different significance with respect to various applications and functionality^36–38^. Therefore, edge strength/weight is a crucial parameter that contributes to identifying the superspreading nodes in complex human interaction networks.

The following are the observations from the above studies with respect to contagious disease spread: firstly, the network considered for the study is not the human interaction network; it has been studied in detail that, for the disease epidemiology, human interaction networks^19,39^ plays a greater significance compared to the other types of the complex networks. Secondly, the majority of super-spreader identification techniques rely on unweighted networks, where all edges are considered equal or assigned a default weight, denoted as $W_{{v_i}{v_j}} = 1$. Thirdly, in the previous studies on weighted networks, where the weights mostly did not capture the interaction duration between the contacts, either the weights are generated randomly or by using centrality measures. Previous research on the spread of diseases has explored weighted networks, such as transportation networks (e.g., airport networks), where factors like passenger count are taken into account as potential edge weights^40^. The other types of the networks^41^ considered are the scale-Free, Erdös-Rényi, etc., where random weights are used. Weights generated from the centrality measures like degree and k-shell directly influence the network connectivity structure but not contributing to the actual measures like proximity or time spent or contact duration between individuals, etc. The primary limitation of these existing methods is their inability to represent complex relations, such as infection probability rates. Hence, these approaches are not robust to anticipate disease occurrence or its arrival times. Mainly in the spread of contagion, the infection starts propagating from one node to the other nodes via its edges^19,42,43^. Insights from the studies^21,22,42,43^, we attempted to obtain the interaction duration as edge weights (Please refer to Algorithm 1) to analyze its robustness in ranking superspreading nodes more optimally in the context of contagious disease spread. In the current study, assuming all the adjacent nodes are in proximity. We use total contact duration/interaction time as the derived edge weights between pair of nodes or contacts.

**Fig. 1:**
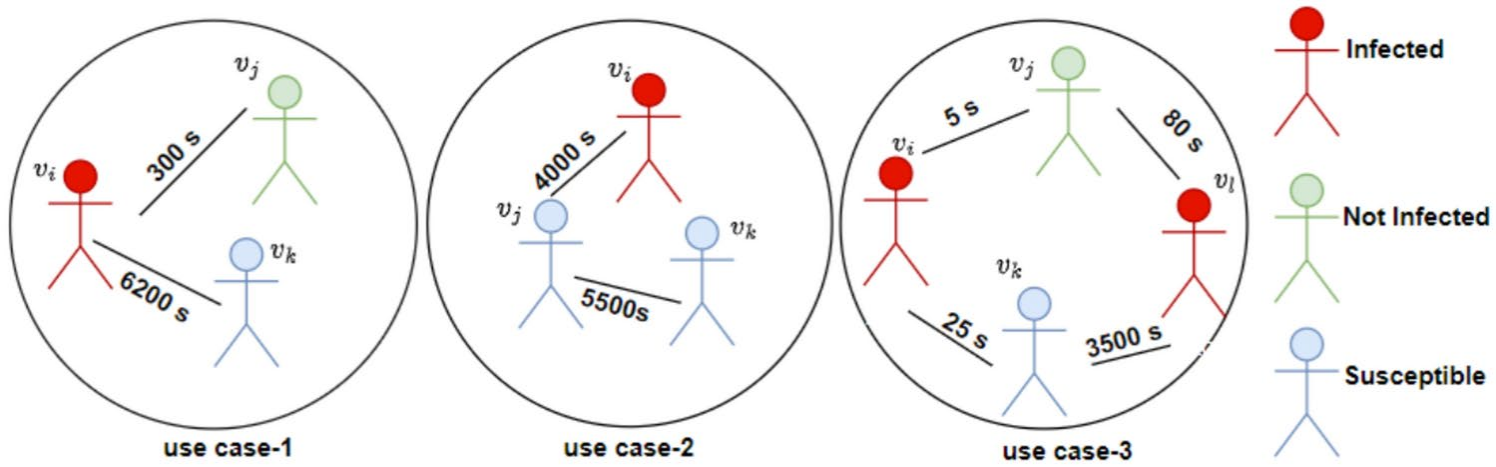
Scenarios demonstrating the importance of the interaction duration for contagious disease spread.

**Fig. 2:**
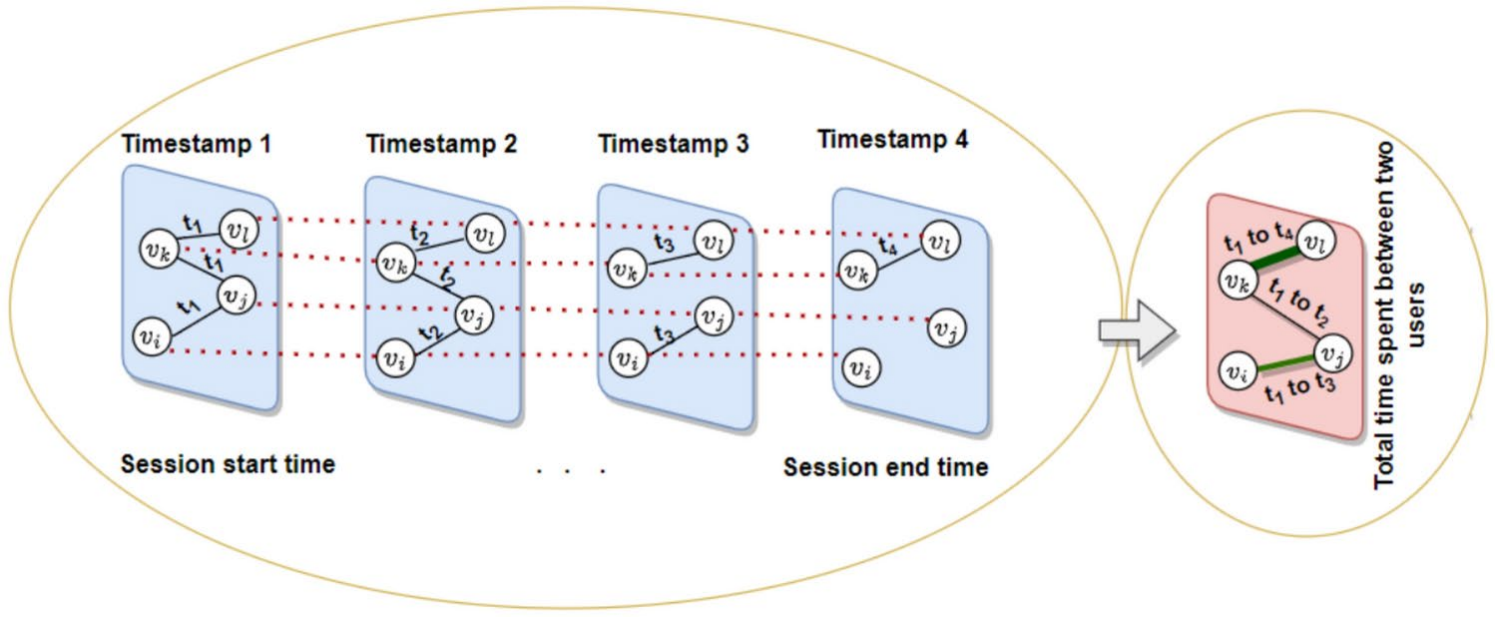
Analysis of contact network for time-based weight estimation. Total amount of contact time between individuals where timestamp range from [$t_1$ to $t_4$]. The thicker line indicates larger amount of time spent between individuals.

In Fig. 1, we show the significant use cases elaborating the need of modeling edges and their weights in contact networks. Use case-1: Suppose two individuals are interacting, where user $v_i$ is infected and is in contact with user $v_j$, but their contact time is minimal. Even though user $v_j$ is in contact with infected user $v_i$, user $v_j$ may not be infected due to less contact time with user $v_i$, or user $v_j$ might have a strong immune system. But on the other hand, user pairs ($v_i$, $v_k$) have longer contact time; due to this, there is a high possibility that $v_k$ is infected by $v_i$. In summary, longer contact duration’s significantly raise the risk of transmission, highlighting the critical role of contact duration in spreading the infection between pairs of contacts. Here, infection spreads directly from the infected nodes to its direct contact. Use case-2: user ($v_i$, $v_j$) has a large contact duration; hence $v_j$ is infected by $v_i$, there is contact between pairs ($v_j$, $v_k$) for a longer duration of time, hence $v_k$ is infected by $v_i$ through $v_j$. In summary, the infection will spread from one user to another user, not only from direct contacts of the infected user but also from indirect contacts. Use case-3: user $v_i$ and user $v_j$’s interaction time is minimal, and user $v_i$ and $v_k$ interaction time are also minimal. As a result, both $v_j$ and $v_k$ are not infected by user $v_i$. But the contact duration between ($v_k$, $v_l$) is more; hence $v_k$ is infected by $v_l$ (not by node $v_i$). The contacts between pairs ($v_j$, $v_l$) is minimal, hence $v_j$ is not infected by $v_l$. Therefore, the superspreading node in this use case is $v_l$, not $v_i$. In summary, use case-3 highlights how contact durations directly influence infection probabilities, with both minimal and extended durations playing crucial roles in determining infection transmission within the network. Finally, the temporal information is considered to be the primary requirement because infection propagation is directly proportional to the contact time. Therefore, ranking the node or identifying the superspreading node requires estimating the interaction duration between contacts, which differs from the edge weights derived from the indices of network properties, such as the degree or the k-shell value. Therefore, our proposed method detects the superspreading node and effectively assigns distinct ranks or influence scores to individual nodes. This is determined by considering the network structure and edge weights, derived from the interaction time of contacts in human contact networks.

## Proposed method

This work is inspired by the studies^40,44,45^, which shows the importance of the weighted network and the crucial parameters to be considered for studying real complex networks in the case of epidemiological analysis. In this work, we have derived edge weights from the human contact networks in terms of the amount of time spent between two individuals or connected pairs of nodes, which are helpful in epidemiological studies, especially in the case of contagion spread. Here, we propose a technique called Real Weighted Influence (*RWInf*) which takes interaction duration as edge weights and leverages network local and global properties to rank the superspreading nodes in the real human interaction networks.

### Estimating temporal statistics

In this study, first we have modeled the graph as an undirected weighted network, *G*(*V*, *E*, *W*) where |*V*| represents the vertex set, |*E*| represents the edge set, and *W* is the weights in each edge such that $\{w_1,w_2,w_3,....,w_{|E|}\}$. Our aim here is to identify the time spent between the contacts and then to utilize this quantity as the edge weight, as shown in Fig. 2. During the contact between two individuals, they may be in contact for a certain duration, then they disconnect. Further, they might be in contact with other users. The same scenario is depicted in the Fig. 2. Finally, creating the graph as a static weighted network where each edge is associated with the weight attribute.

This study is mainly helpful for understanding important epidemiological parameters such as contact duration to identify influential contacts and preserve the properties of the dynamic contact networks^46^. The process of computing session start-time ($time_j$) and session end-time ($time_i$) between contacts/individuals is given in Algorithm 1. After deriving the session start-time (*st*) and end-time (*et*), we need to compute the length of stay between two individuals called connection strength or edge weight ($W_{{v_i}{v_j}}$), as follows:

11 $$\begin{aligned} W_{{v_i}{v_j}} = \sum _f(et_{v_i v_j} - st_{v_i v_j}) \end{aligned}$$

where *f* represents the number of times two users interact, the start-time and end-time of each interaction between the individuals are indicated by $st_{v_i v_j}$ and $et_{v_i v_j}$. The connection strength ($W_{{v_i}{v_j}}$), which we considered as the actual edge weights for further analysis. In Fig. 2, the edge between pairs ($v_k, v_l$) is thicker, representing a longer contact time compared to the pairs ($v_i, v_j$) and ($v_j, v_k$).

**Fig. 3:**
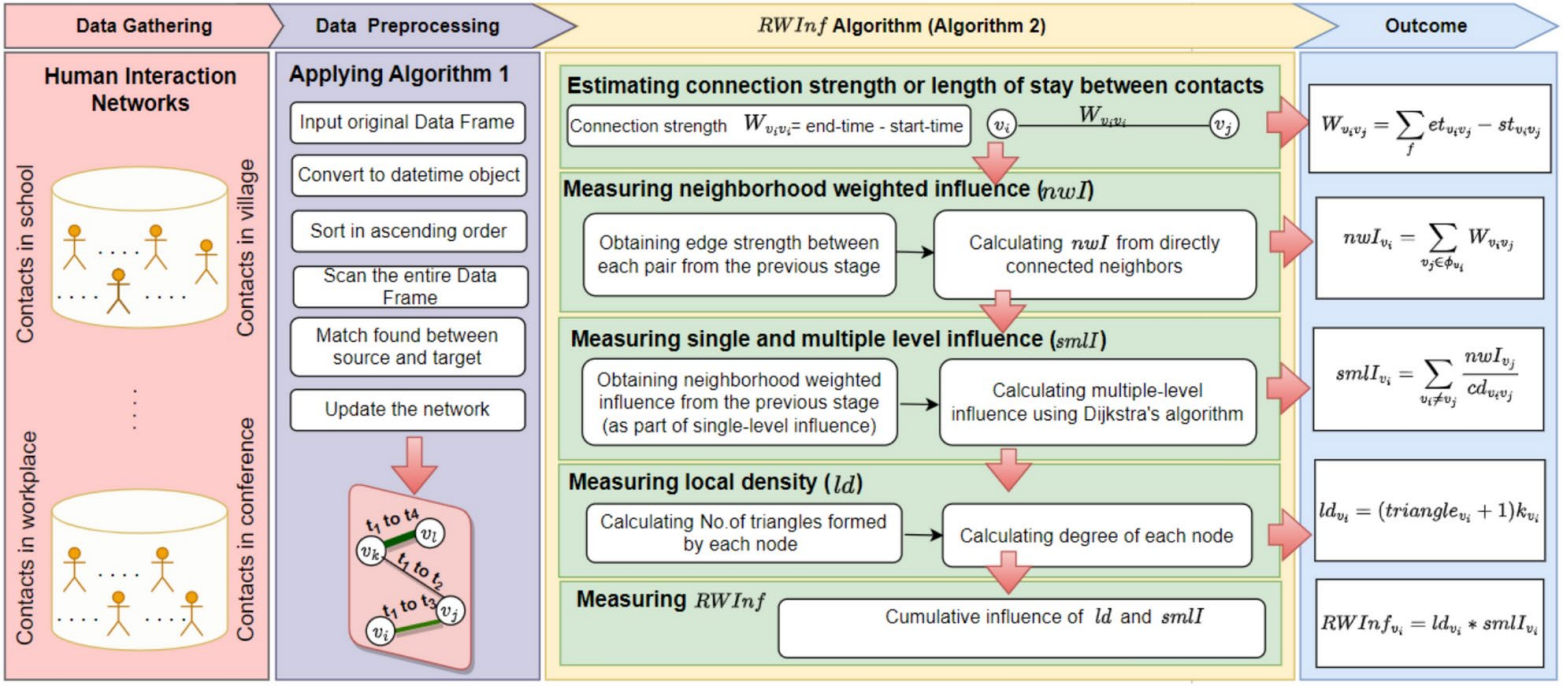
Process flow of *RWInf* by utilizing human interaction networks.

**Algorithm 1:**
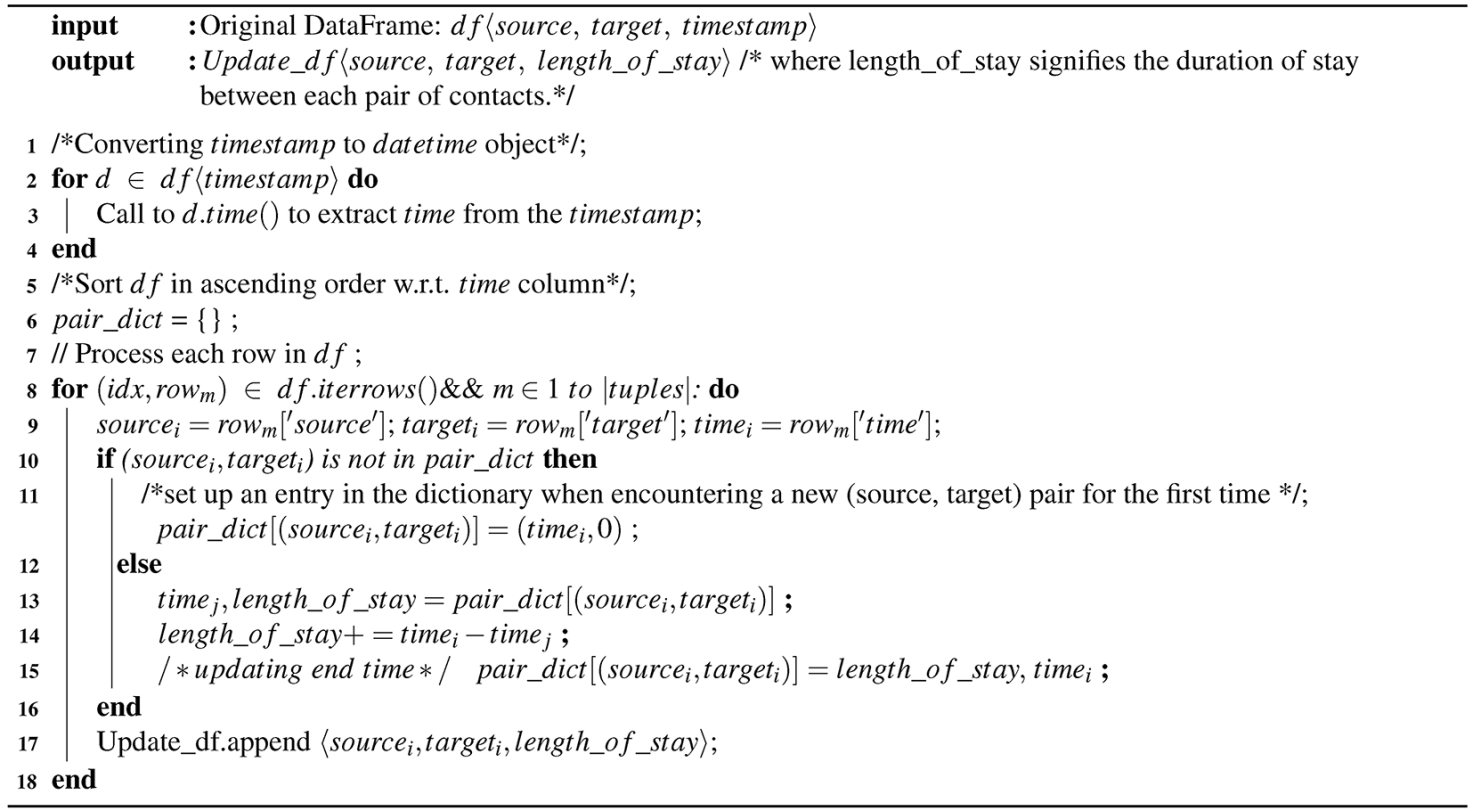
Computing interaction duration between contacts.

### Neighborhood weighted Influence (*nwI*)

Once we obtain the connection strength between two individuals as an edge attribute, it is essential to quantify how this quantity influences the neighboring nodes. The stronger the connection strength signifies the high possibility of contagion spreading towards the neighboring contacts and vice versa. In this case, we are leveraging not only the neighborhood network structure from the directly connected neighbors but also the connection strength. Hence, we identify whether the neighboring nodes will propagate its infection to their second-level neighbors or not. Computing the power of propagation during contagion spread is formulated as follows:

12 $$\begin{aligned} nwI_{v_i} = \sum _{v_j \in \phi _{v_i}} W_{{v_i}{v_j}} \end{aligned}$$

where $nwI_{v_i}$ represents neighborhood weighted influence (*nwI*) of a node $v_i$, $W_{{v_i}{v_j}}$ represents connection strength between nodes $v_i$ and $v_j$, and $\phi _{v_i}$ represents the $v_i$’s neighbors.

### Single and Multiple Level Influence (*smlI*)

Neighborhood weighted influence (*nwI*) is considered as part of the local influence (single-level influence) because this takes into account only the 1-hop neighborhood structure. As part of the multiple-level influence (global influence)^47^, we are using the distance measure called contact distance ($cd_{v_i v_j}$) which will be computed using Dijkstra’s algorithm is the global shortest distance from node i to j, so it could represent the contact distance and the “level” here. Hence, $cd_{v_i v_j}$ will be helpful for obtaining entire global information about the network or the location status of the different individuals in terms of contact distance. Finally, the impact of the node influence is the cumulative influence between direct neighbors and neighbors from different levels of the network. Furthermore, node influence is inversely proportional to the contact distance between individuals who are in contact, and it is formulated as follows:

13 $$\begin{aligned} smlI_{v_i} = \sum _{v_i \ne v_j} \frac{nwI_{v_j}}{cd_{v_i v_j}} \end{aligned}$$

### Local density (*ld*)

This metric obtains information about how strongly the neighborhood nodes are connected. This can be well captured by using degree (k) measure. The degree is the local measure that captures the local structure of the network. In case of infectious disease spread, degree measure is helpful to identify the number of individuals in direct contact with the infected individuals. Further, calculating the number of triangles^27^ helps to gather important information about whether the directly connected neighbors of an infected contact are neighbors among themselves or not. It also reveals the connectedness property of the directly connected neighbors. Degree centrality only collects data from its immediate neighbors, but the triangular structure-property of the node can reveal whether those neighbors are in contact. Hence, the probability of infection propagation among the three neighbors is well determined. This provides significant information within the neighborhoods called local density, and it is formulated as follows:

14 $$\begin{aligned} ld_{v_i} = (triangle_{v_i}+1)k_{v_i} \end{aligned}$$

where the degree of the node *i* is represented as $k_{v_i}$, and ($triangle_{v_i}+1$) signifies that there might be zero triangles existing as mentioned in the study^48^. In such a case, the number of triangle counts may be zero with respect to some nodes. To work well with both large and small-scale networks, we are generalizing the formula by assuming that at least one triangle exists for each node. Both triangle and degree are helpful in obtaining the local density of the neighborhood, hence they are proportional to each other.

### Real weighted Influence (*RWInf*)

In this metric, the *ld* and *smlI* are combined to formulate the *RWInf* (pseudocode is given in Algorithm 2), as follows:

15 $$\begin{aligned} \begin{aligned} RWInf_{v_i} = ld_{v_i} * smlI_{v_i} \\&\hspace{-23mm} = [(triangle_{v_i}+1)k_{v_i}] * \sum _{v_i \ne v_j} \frac{nwI_{v_j}}{cd_{v_i v_j}} \end{aligned} \end{aligned}$$

The cumulative score is the multiplication of *ld* and *smlI* because the node’s influence is directly proportional to the local structure and its global property of the network. The overall flow of the *RWInf* is presented in the Fig. 3.

**Fig. 4:**
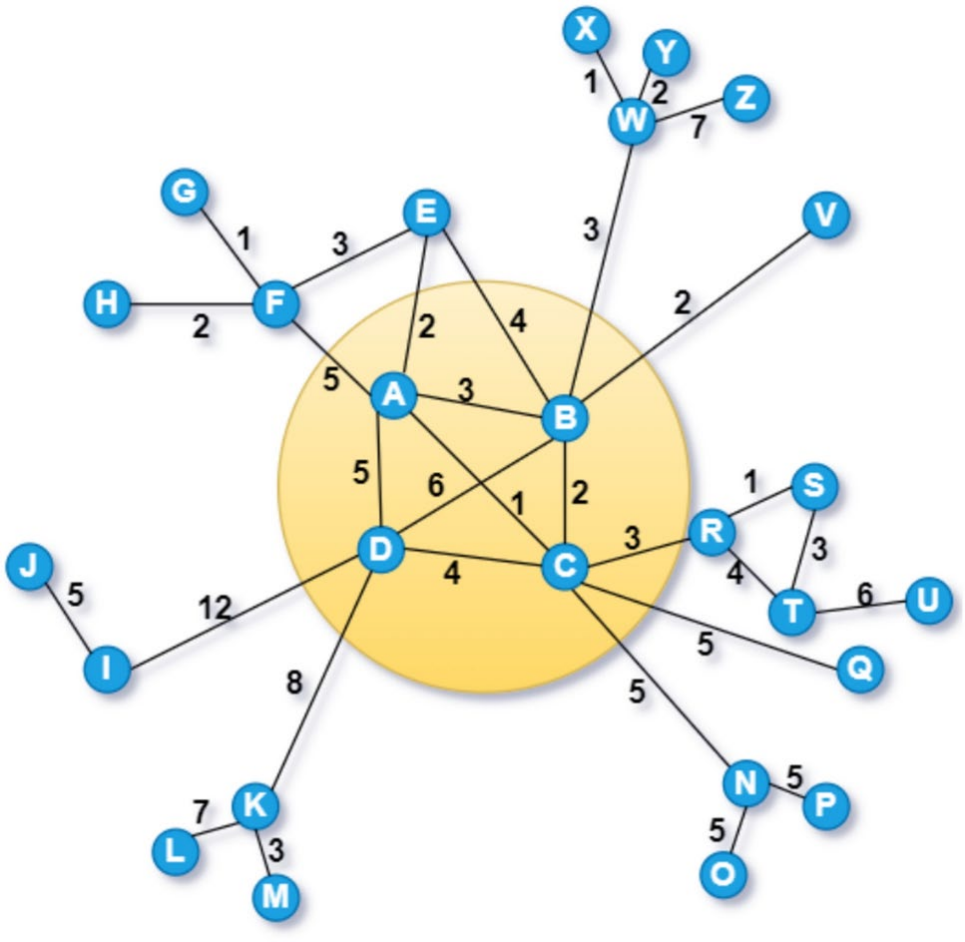
Toy network consisting of nodes connection structure, and the connection strength, where connection strength/edge weights used in this toy network are the hypothetical values used for calculating *RWInf*. Nodes that are present inside the circle are the core nodes.

### *RWInf* score computation

Consider the toy network in Fig. 4, where values on the edge represent the hypothetical edge weight values, representing the time spent between two individuals as part of the stage 1 of the proposed framework. In the case of real networks used for the experimental study, where weights are derived from the Algorithm 1 to extract interaction time between pair of contacts. The rationale behind this work is that the edge weight potential in terms of interaction time is more effective than the edge weight derived from the degree or the k-shell value. Hence our proposed method is successful for the optimal identification of superspreader. In stage 2, we need to calculate neighborhood weighted influence (*nwI*) using Eq. (12) and the computed values for the nodes are as follows: $A = 16$, $B = 20$, $C = 20$, $D = 35$, $E = 9$, $F = 11$. $G = 1$, $H = 2$, $I = 17$, $J = 5$, $K = 18$, $L = 7$, $M = 3$, $N = 15$, $O = 5$, $P = 5$, $Q = 5$, $R = 8$, $S = 4$, $T = 13$, $U = 6$, $V = 2$, $W = 13$, $X = 1$, $Y = 2$, $Z = 7$. In stage 3, we need to estimate the single and multiple-level influence (*smlI*) for each of the nodes using Eq. (13) as follows:

$$\begin{aligned} \begin{aligned} smlI_A =&\frac{nwI_B}{cd_{AB}} + \frac{nwwI_C}{cd_{AC}} + \frac{nwI_D}{cd_{AD}} + \frac{nwI_E}{cd_{AE}} + \frac{nwI_F}{cd_{AF}} + \frac{nwI_G}{cd_{AG}} + \frac{nwI_H}{cd_{AH}} + \frac{nwI_I}{cd_{AI}} + \frac{nwI_J}{cd_{AJ}} + \frac{nwI_K}{cd_{AK}} + \frac{nwI_L}{cd_{AL}} + \frac{nwI_M}{cd_{AM}} + \frac{nwI_N}{cd_{AN}} + \frac{nwI_O}{cd_{AO}} \\&+ \frac{nwI_P}{cd_{AP}} + \frac{nwI_Q}{cd_{AQ}} + \frac{nwI_R}{cd_{AR}} + \frac{nwI_S}{cd_{AS}} + \frac{nwI_T}{cd_{AT}} + \frac{nwI_U}{cd_{AU}} + \frac{nwI_V}{cd_{AV}}+ \frac{nwI_W}{cd_{AW}} + \frac{nwI_X}{cd_{AX}} + \frac{nwI_Y}{cd_{AY}} + \frac{nwI_Z}{cd_{AZ}}\\&= \frac{20}{1} + \frac{20}{1} + \frac{35}{1} + \frac{9}{1} + \frac{11}{1} + \frac{1}{2} + \frac{2}{2} + \frac{17}{2} + \frac{5}{3} + \frac{18}{2} + \frac{7}{3} + \frac{3}{3} + \frac{15}{2} + \frac{5}{3} + \frac{5}{3} + \frac{5}{2} + \frac{8}{2} + \frac{4}{3} + \frac{13}{3} + \frac{6}{4} + \frac{2}{2} + \frac{13}{2} + \frac{1}{3} \\&+ \frac{2}{3} + \frac{7}{3}\\&= 154.333 \end{aligned} \end{aligned}$$

Then, determining local density (*ld*) score in stage 4, using Eq. (14) where degree of node *A*, $k_A = 5$ and $(triangle_A +1) = 6$; $ld_A = 5 * 6 = 30$. In stage 5, *RWInf* is computed using Eq. (15) as follows:

$$\begin{aligned} \begin{aligned} RWInf(A) = 154.333 * 30 = 4629.99 \end{aligned} \end{aligned}$$

The influence scores of the remaining nodes (B to Z) are computed similarly. In conclusion, nodes receive rankings determined by their influence scores. A higher score corresponds to greater influence, and it is ranked as one.

**Algorithm 2:**
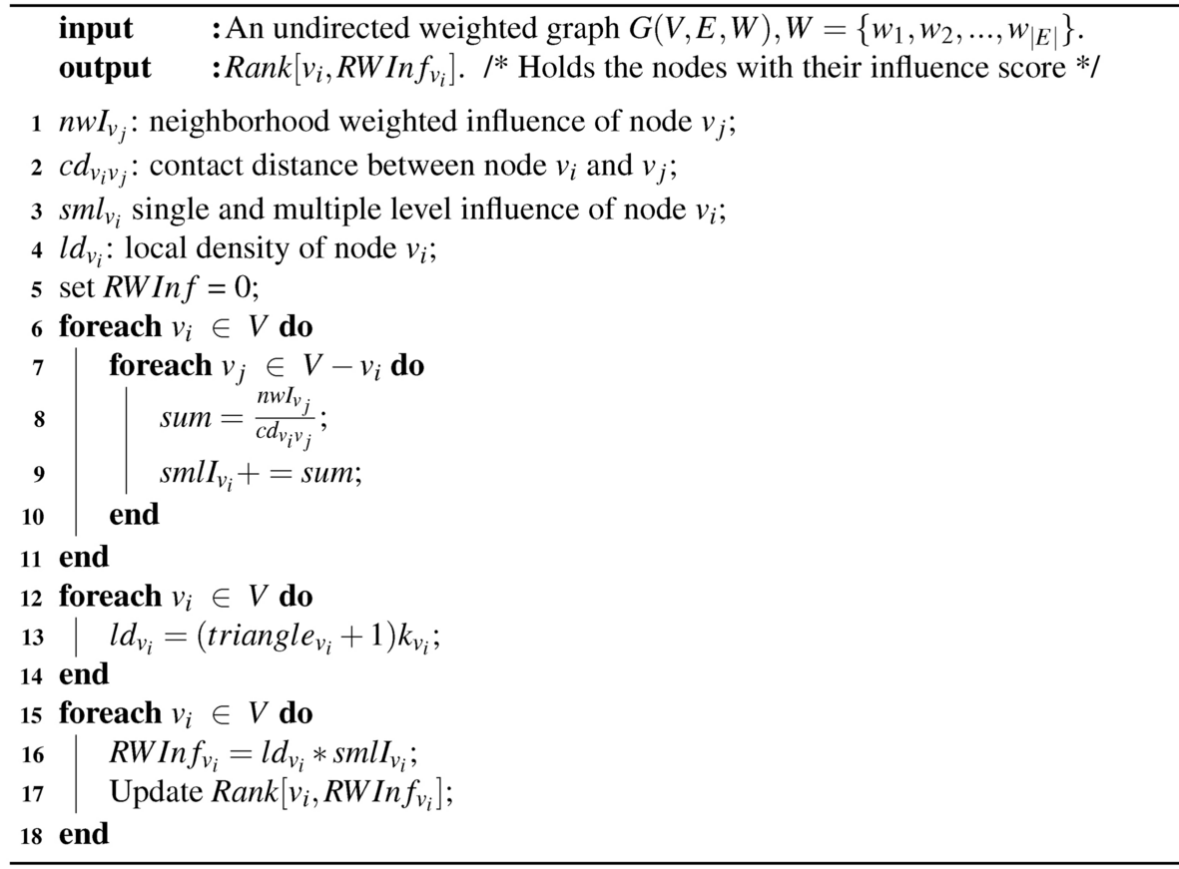
RWInf: Node ranking in the weighted network

### Complexity analysis of RWInf

Computing the neighborhood weighted influence ($nwI$) for all nodes requires iterating over all edges in $E$, resulting in a time complexity of $O(E)$. The $smlI$ measures global influence, and since it uses Dijkstra’s algorithm for all pairs, its time complexity is $O(V^2 + E \log V^2)$. After calculating the degree and triangle count for each node, the computation of local density ($ld$) for all nodes takes $O(V)$. Considering all the steps, the dominating factor in the overall time complexity is $O(V^2 + E \log V^2)$. The overall effectiveness of *RWInf* is comparable with the baseline approaches like $C_{B} ^ W$ and $C_{C} ^ W$. The algorithm is scalable as long as the edge weights, which represent contact duration, are easily accessible to the RWInf algorithm. Hence, the time complexity of *RWInf* is better.

## Experimental setup

In this section, we broadly describe the datasets used for our study, the comparison algorithm, and details about the evaluation metrics, such as kendall’s correlation coefficient and susceptible-infected-recovered (SIR) model.

### Datasets

Sociopatterns datasets^49^ are used in this study are the contact networks. It shows the contacts who are active during 20-second data collection intervals. We have used six datasets in total, and the dataset statistics are presented in Table 1. These datasets are the contact networks wherein the contact information is captured using sensor technology and are used in most of the epidemiology studies^50,51^. The primary goal of this research is to create a generalized algorithm that effectively leverages on both derived weights from the contact duration and/or predefined weights specified in the dataset. For the first five datasets in Table 1, the dataset parameters are available in the following format: (source, target, timestamp), where we can derive the weights based on their interaction time and the frequency of interaction if there are repetitions of the same (source, target) pairs. Using Algorithm 1, we extracted the total length of stay between each pair of connected individuals. Then, we considered this contact duration information as the edge weights to form the weighted networks and it is shown in^52^. On the other hand, the sixth dataset is mentioned in Table 1, where parameters are available in the following format: (source, target, weight) where the weight information is already in the dataset only. The assigned threshold value: (i) weight = 1 if the contact duration is less than 5 minutes, (ii) weight = 2 if the contact duration is between 5 and 15 min, (iii) weight = 3 if the contact duration is between 15 min and 1 h, (iv) weight = 4 if the contact duration is more than 1 h. Therefore, it is important to note that the weights are already predefined in the sixth dataset based on contact duration, and we have utilized the same in our work. This set-up will help to validate the proposed algorithm efficacy on an assigned threshold value. Finally, the empirical results presented in the Results section prove that our proposed approach outperforms the existing indices for predefined as well as derived weights, concluding the generalizability of the proposed approach.

**Table 1:** Properties of the human contact networks, *V*: vertex set, *E*: edge set, $\beta _{th}$: epidemic threshold, $\beta$: infection probability, *D*: network diameter, $\langle k \rangle$: average degree, P$\langle mean \rangle$: average shortest path length, and *C*: average clustering coefficient.

S. No	Dataset	|*V*|	|*E*| (frequency of contacts)	$\beta _{th}$	$\beta$	*D*	$\langle k \rangle$	P$\langle mean \rangle$	*C*
1	SFHH^53^	403	70261	0.015	0.05	4	24.538	1.953	0.282
2	Workplace-1^54^	217	78249	0.022	0.06	5	19.696	1.882	0.387
3	Workplace-2^55^	92	9827	0.050	0.1	3	8.207	1.964	0.426
4	Primary school^56^	242	125773	0.013	0.05	3	34.368	1.732	0.526
5	High school-1^57^	180	45047	0.034	0.07	4	12.439	2.147	0.475
6	High school-2^58^	120	502	0.148	0.2	12	4.183	5.362	0.458

### Comparison algorithms

Here, we discuss some empirical settings we have used while comparing the baseline algorithms with our proposed algorithm *RWInf*.

- Baseline Algorithms: The first baseline algorithm we considered is $W_{k-shell}$^28^. In this work^28^ real-weighted networks are used such as the US air transportation network, corporate ownership network. In those networks, weights are already defined, and the same quantities are utilized in $W_{k-shell}$. In our study, the $W_{k-shell}$ algorithm has been applied with weights (i.e., interaction duration) derived from the contact networks. In the case of other baseline algorithms, i.e., *Wks*, $ksd^w$, *ngsc*, and *HIC* algorithms, we follow the respective baseline methodology for the weight generation (using either k-shell or degree, etc.).
- State-of-the-art approach: For the validation purpose, we have used a weighted version of the degree centrality^31^,^32^, betweenness centrality^31,34^, and closeness centrality^31,34,35^. Since these methods do not explicitly consider edge weights, the weights generated from the Algorithm 1 are used in each of the above algorithms.

### SIR model

The eight comparison algorithms and the proposed *RWInf* use the SIR model score as their ground truth. The SIR model is an epidemic model, which goes through three stages of the node: Susceptible, Infected and Recovered. Initially, we consider a single node serving as the source of infection, with the infection probability $\beta$. The infection spreads from a single node to its neighbors and subsequent levels. If the $\beta$ value is higher, the disease spreading rate is high and vice versa. Therefore, it is essential to compute the optimal values of the $\beta$ with the help of epidemic threshold (i.e., $\beta > \beta _{th}$): $\beta _{th}\sim \langle k \rangle / \langle k^2 \rangle$, where, $\langle k \rangle$: average degree, $\langle k^2 \rangle$: second-order average degree^59^. At each time step *t*, the individual who is infected transitions to the recovery stage, and this transition occurs with a probability rate of $\lambda = 1$. Finally, the spreading process ends when no more individuals are left infected. In the end, the count of recovered individuals is used to compute the individual’s influence score. Further, the series of steps are repeated to have its influence score over 1000 runs on the SIR model.

**Fig. 5:**
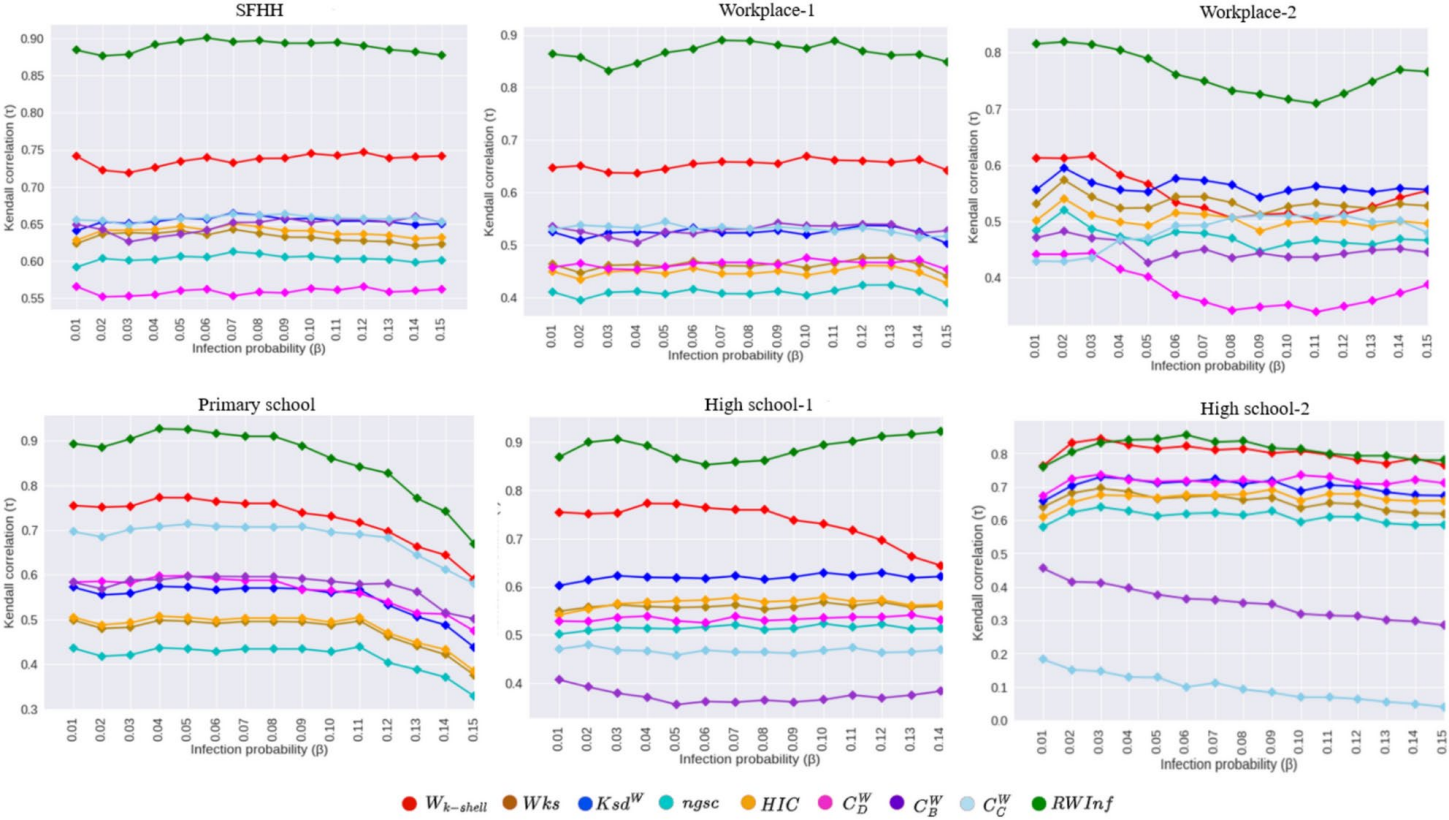
The empirical examination of the data illustrates kendall’s $\tau$ correlation between various comparison methods, including *RWInf* and the SIR scores on several real-world contact networks, where *X*-axis denotes varying $\beta$ values, and *Y*-axis denotes kendall’s $\tau$ correlation score.

**Table 2:** Average score of Kendall’s $\tau$ correlation with varying infection probability rate ($\beta$) from [0.01, 0.15].

Network	$\sigma (\tau _{W_{k-shell}})$	$\sigma (\tau _{Wks})$	$\sigma (\tau _{ksd^w})$	$\sigma (\tau _{ngsc})$	$\sigma (\tau _{HIC})$	$\sigma (\tau _{C_{D}^W})$	$\sigma (\tau _{C_{B} ^ W})$	$\sigma (\tau _{C_{C}^W})$	$\sigma (\tau _{RWInf})$
SFHH	0.736	0.632	0.654	0.603	0.639	0.559	0.647	0.657	0.889
Workplace-1	0.653	0.461	0.524	0.409	0.447	0.463	0.530	0.528	0.867
Workplace-2	0.547	0.533	0.561	0.472	0.503	0.381	0.450	0.483	0.763
Primary school	0.724	0.474	0.546	0.415	0.482	0.562	0.575	0.682	0.857
High school-1	0.724	0.560	0.620	0.515	0.566	0.533	0.374	0.467	0.890
High school-2	0.801	0.655	0.700	0.609	0.665	0.716	0.353	0.098	0.811

**Table 3:** Improvement (in %) of *RWInf* in comparison to different centrality measures with the varying infection probability ($\beta$).

Algorithms	SFHH				Workplace-2				High school-1			
	$\beta =0.01$	$\beta =0.05$	$\beta =0.1$	$\beta =0.15$	$\beta =0.01$	$\beta =0.05$	$\beta =0.1$	$\beta =0.15$	$\beta =0.01$	$\beta =0.05$	$\beta =0.1$	$\beta =0.15$
$W_{k-shell}$	19.287	22.018	19.974	18.312	33.008	39.241	39.227	38.005	15.155	12.196	22.481	56.488
*Wks*	41.772	39.834	41.341	40.847	53.528	50.535	36.060	45.123	58.304	55.575	57.473	62.343
$ksd^w$	37.918	36.241	35.870	34.921	46.613	42.698	42.698	37.608	44.257	40.024	42.125	47.068
*ngsc*	49.322	47.805	47.285	45.995	68.606	70.156	55.788	64.220	73.281	69.047	70.812	77.454
*HIC*	40.761	38.552	39.433	38.839	62.591	60.158	44.009	54.326	60.395	51.819	54.755	62.543
$C_{D}^W$	56.277	59.908	58.593	56.077	84.543	96.208	103.568	97.613	64.362	63.775	67.174	72.248
$C_{B} ^ W$	36.165	40.903	36.968	34.526	73.003	84.977	64.072	72.086	113.449	143.623	144.634	139.183
$C_{C}^W$	34.873	36.312	35.497	34.423	89.682	67.850	40.529	59.750	84.565	89.009	91.128	96.192

**Fig. 6:**
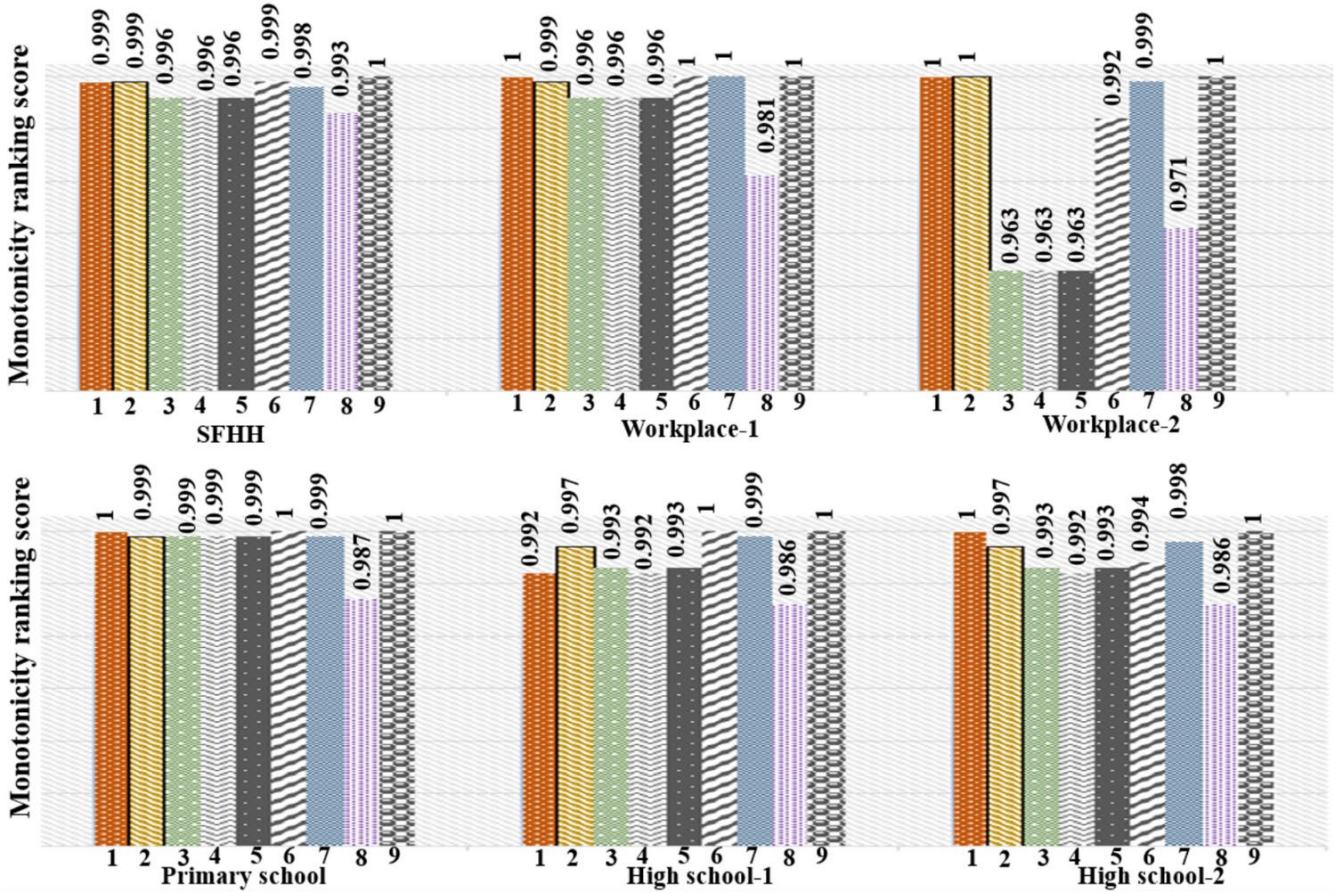
Ranking uniqueness of 8 comparison algorithms and proposed approach *RWInf*. Numbering on the X-axis represents different indexing approaches, where 1: $W_{k-shell}$, 2: *Wks*, 3: $ksd^w$, 4: *ngsc*, 5: *HIC*, 6: $C_{D}^W$, 7: $C_{B} ^ W$, 8: $C_{C}^W$, 9: *RWInf*.

### Kendall’s correlation coefficient

Once we obtain the influence score from all the comparison algorithms, the proposed approach *RWInf*, and the SIR model, we need to assign ranks to each node in the network based on its influence score. The top-1 rank is given to such node, which will have the maximum influence score, and the least rank is assigned to those nodes whose influence score is minimum. After ranking the nodes, the correlation between each comparison algorithm and the SIR model score is examined. If the two ranking lists are highly correlated, then kendall’s $\tau$ correlation score is closer to 1; otherwise, it is closer to $-1$^60^.

## Results and discussion

Here, we demonstrate the set of experiments we have carried out to analyze the robustness of the proposed approach.

### Effectiveness of *RWInf* in terms of spreading dynamics

This analysis is conducted to estimate kendall’s $\tau$ correlation coefficient as it is an important measure to identify the ranking correlation between each of the comparison algorithms (eight existing algorithms, proposed *RWInf*) and the SIR model, and it is formulated as follows:

16 $$\begin{aligned} \sigma (\tau )=\frac{\sum ^{iter}_{i=1} \tau (\beta _{th}+0.01* i)}{iter}. \ \end{aligned}$$

where, *iter* denotes the number of iterations, and we have set $iter = 15$. During each iteration the $\beta$ is incremented by 0.01.

Figure 5 demonstrates the effectiveness of the proposed approach *RWInf* over eight comparison algorithms. The observations are as follows: (1) we have considered two setups of the networks to examine the robustness of the *RWInf*, in both cases *RWInf* performs well compared to the other baseline algorithms. The study^4^ also states the importance of considering edge weights in the form of interaction duration. From the empirical analysis, we conclude that our approach outperforms by considering edge weights in terms of interaction duration or setting up the threshold during weight assignment. (2) we have also observed that computing interaction duration and incorporating them in the existing algorithm also plays a major role. For example, the first baseline algorithm we have considered for the comparison is $W_{k-shell}$^28^. We have employed the $W_{k-shell}$ algorithm by incorporating the edge weights computed from our current study. Hence, the analysis present in Fig. 5 and Table 2 shows that $W_{k-shell}$ will be the second effective algorithm for the datasets SFHH, Workplace-1, Primary school, High school-1, and High school-2 networks; (3) most of the baseline algorithms, such as *Wks*, $ksd^w$, *ngsc*, and *HIC* utilize k-shell or degree measures for estimating the edge weight; this may be one of the significant reasons for the low performance of these algorithms on the contact networks; (4) weighted degree, betweenness, and closeness centrality show average performance on all the datasets.

### Measuring ranking uniqueness

Measuring ranking uniqueness is broadly studied as monotonicity ranking^9^. Each individual in the network will have a varying connection structure, and the edge attributes, such as contact duration time, may also differ. These properties of the networks signify the uniqueness of the individual influence, and it is measured as follows:

17 $$\begin{aligned} M(I) =\Big [1-\frac{\sum ^V_{i\in I} V_i(V_i-1)}{V(V-1)}\Big ] ^2. \ \end{aligned}$$

Where, *V* denotes the total number of individuals in the network, $V_i$ signifies the node set with a similar score of *i*, and *I* represents the indexing approach. The value of the monotonicity (*M*(*I*)) varies between 0 (similar ranking) to 1 (unique ranking). Figure 6 shows the visualization of the monotonicity on the entire network. Out of eight comparison algorithms, other baseline algorithms provide a unique ranking to some of the networks, but *RWInf* provides a unique ranking for all the networks.

### Measuring improvement percentage of *RWInf* over comparison algorithms

**Fig. 7:**
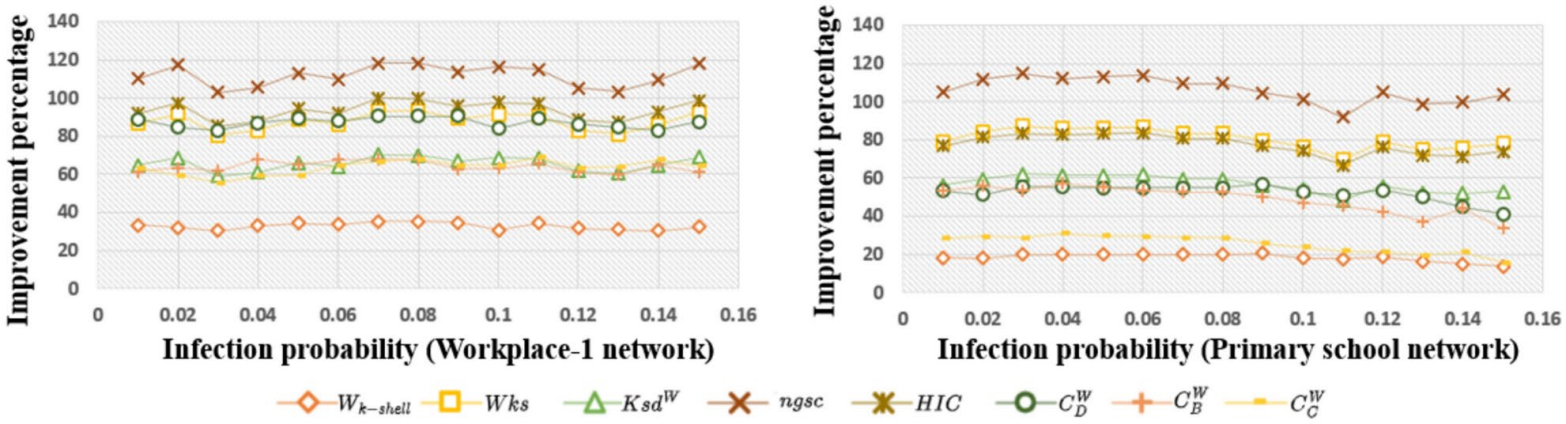
Visualizing percentage improvement of Workplace-1 and Primary school networks.

Percentage improvement, i.e., $\eta (\%)$^61^ among the indexing approaches are calculated using the following models:

18 $$\begin{aligned} \eta _I(\%)={\left\{ \begin{array}{ll} \frac{\tau _C(I) -\tau _I}{\tau _I} * 100, & \tau _I>0\\ \frac{\tau _C(I) -\tau _I}{-\tau _I} * 100, & \tau _I<0\\ 0, & \tau _I=0 \end{array}\right. } \end{aligned}$$

where, $\tau _C(I)$ indicates the rank correlation between *RWInf* and the SIR model, and $\tau _I$ shows the rank correlation between the comparison algorithms and the SIR spreading. Each $\eta$ score has a different significance, such as $\eta < 0$ signifies that the proposed method performance is worse than the comparison algorithms. Similarly, $\eta > 0$ signifies that the performance of the proposed approach is better, and $\eta = 0$ means that there is no improvement. Table 3 shows the improvement percentage of the contact networks SFHH, Workplace-2, and High school-1 with the varying infection probability rate as 0.01, 0.05, 0.1, and 0.15. The $\eta (\%)$ for the *C*(*Wks*), $C(ksd^w)$, *C*(*ngsc*), and *C*(*HIC*) over *Wks*, $ksd^w$, *ngsc* and *HIC* are relatively greater for all the networks. The graphical visualization of the improvement percentage of each of the baseline algorithms on the Workplace-1 and Primary school networks is presented in Fig. 7 where $\beta$ ranges from 0.01 to 0.15. The state-of-the-art methods, such as $C(C_{D}^W)$, $C(C_{B} ^ W)$, $C(C_{C}^W)$ over $C_{D}^W$, $C_{B} ^ W$, $C_{C}^W$ also yields better results for all the six complex networks used for our study, where $\beta > \beta _{th}$. Here we presented all the first five network improvement percentages with respect to different indexing measures. Therefore, the above observation shows *RWInf* outperforms the existing indexing measures with respect to $\eta .$

**Fig. 8:**
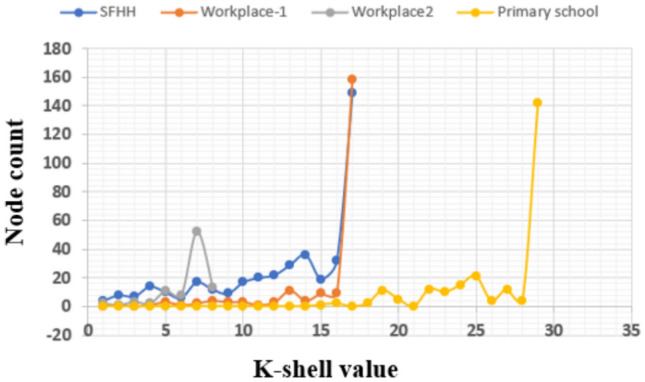
Range of nodes’ k-shell values of different contact networks.

**Fig. 9:**
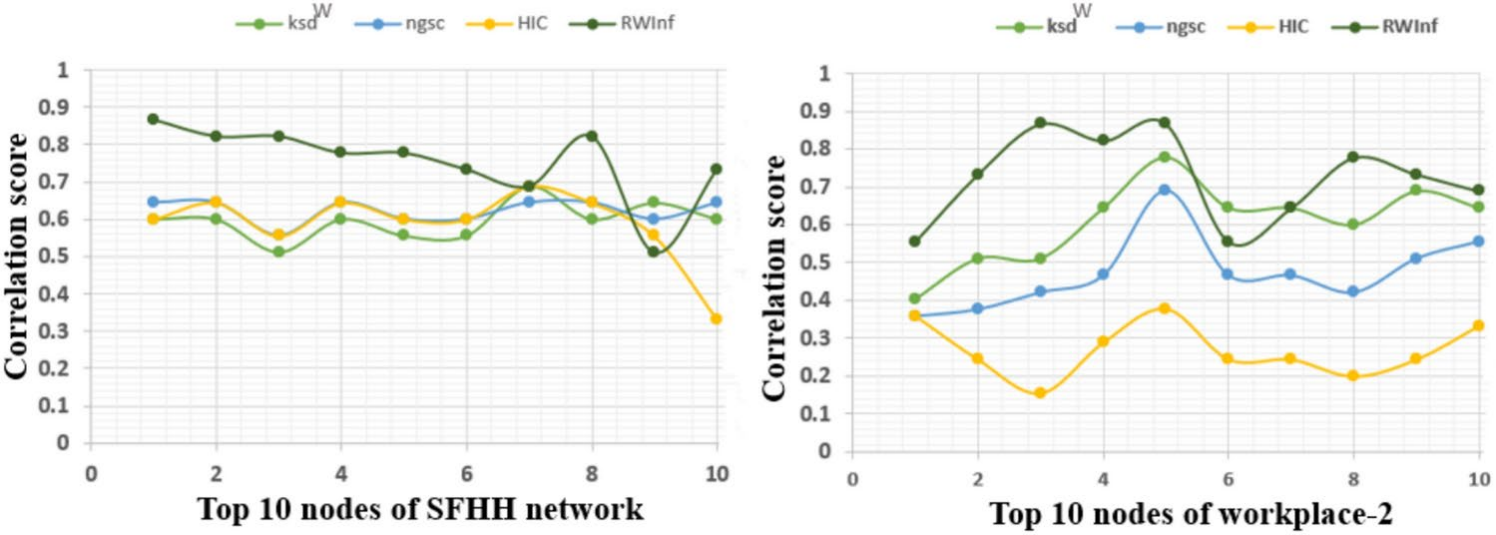
Top 10 nodes where infection probability rate range from 0.01 to 0.1.

From the above empirical analysis, it is clear that the edge weights in terms of interaction duration contribute more while identifying the influential spreader than the weights generated from the k-shell and degree measures. The Fig. 8 shows the distribution of the k-shell values among the number of nodes, where *X*-axis indicates the number of k-shell values present in the SFHH, Workplace-1, Workplace-2, and Primary school contact networks. The *Y*-axis shows the number of nodes that fall under each bucket (ranging from 1 to 35) of the k-shell value. We have examined that most of the nodes are having the same k-shell values, and the same is observed in Fig. 8. When we use the k-shell values for the weight generation, each node pair may not have unique edge weights, but practically when two individual nodes interact together, they may have varying interaction times. Hence, unique weight generation is essential for optimal superspreading node identification, compared to setting the threshold values or weights generated from centrality measures. From these analyses, it is clear that the proposed *RWInf* is an effective approach for identifying superspreading individuals with the help of edge weights in terms of interaction duration between contacts. Moreover, we have also provided the visualization of the Top-10 nodes of *RWInf* in comparison to recent techniques, such as $ksd^w$, *ngsc*, *HIC* (as shown in the Fig. 9), with the infection probability ranging from 0.01 to 0.1. The Top-10 node visualization shows that the SIR model score correlates more with the proposed *RWInf* than more recent algorithms. Finally, we observe that the recent algorithms, such as $ksd^w$, *ngsc*, and *HIC*, are helpful in identifying the influential node in other complex networks such as social networks, citation networks, collaboration networks, neural networks, etc. However, the proposed *RWInf* is more optimal for the infectious disease epidemiology study because of the use of human contact networks and its real-time properties. In addition, the RWInf can be applied directly to practical applications such as design of vaccination or quarantine strategies. RWInf can prioritize vaccination by identifying superspreaders, enabling efficient resource allocation to high-risk individuals or clusters. Early detection of superspreaders through RWInf allows targeted quarantines, minimizing societal disruption and healthcare strain. The algorithm remains scalable if edge weights, representing contact duration, are readily available.

## Conclusions

In this article, we introduce a novel method, *RWInf*, for identifying superspreading nodes. This approach incorporates real weights based on the duration of contact times. We empirically evaluated on real-world contact networks that *RWInf* is a robust influential node detection algorithm compared to the recent algorithms such as $ksd^w$, *ngsc*, and *HIC*. We also showed the performance of *RWInf* on two different set-ups of the networks, considering derived edge weigths from the interaction duration as well as variable threshold levels. Further experiments on contact networks revealed that generating unique edge weights (i.e., interaction duration) plays a vital role in the optimal identification of superspreading nodes than the weights generated by the network metrics (k-shell and degree). We have also verified the interaction duration potential by incorporating these weights in state-of-the-art algorithms, like $C_{D}^W$, $C_{B} ^ W$, and $C_{C}^W$ and observed that deriving optimal edge weight is the necessary requirement to analyze disease pathway. Finally, we have developed the new superspreading node identification algorithm *RWInf* that gives better performance than different comparison algorithms. The algorithm *RWInf* provides a unique ranking addressing the monotonicity ranking issues in the network. The top-ranked nodes selected from the *RWInf* have a greater correlation with the state-of-the-art SIR model. Currently, we have analyzed several real-world datasets to explore their network topology. As part of our future work, we plan to investigate how various network parameters, such as node distribution across networks, density, and others, influence model performance. Additionally, we can incorporate contact duration as real weights to model dynamic temporal networks for influential node detection. We may also use these weights to generate the node embedding as part of the edge feature to identify more prominent nodes using deep learning techniques.

## Data Availability

The datasets generated during and / or analyzed during the current study are made available in the Sociopatterns repository, http://www.sociopatterns.org/datasets/

## References

[1] Riolo, C. S., Koopman, J. S. & Chick, S. E. Methods and measures for the description of epidemiologic contact networks. *J. Urban Health* **78**, 446–457. 10.1093/jurban/78.3.446 (2001). 11564848 10.1093/jurban/78.3.446 PMC3455912

[2] Ozella, L. et al. Using wearable proximity sensors to characterize social contact patterns in a village of rural malawi. *EPJ Data Sci.* **10**, 46. 10.1140/epjds/s13688-021-00302-w (2021).

[3] Huang, W., Kuo, Y.-S., Pannuto, P., Dutta, P. Opo: a wearable sensor for capturing high-fidelity face-to-face interactions. In *Proceedings of the 12th ACM Conference on Embedded Network Sensor Systems* 61–75. 10.1145/2668332.2668338 (2014).

[4] Eames, K., Bansal, S., Frost, S. & Riley, S. Six challenges in measuring contact networks for use in modelling. *Epidemics* **10**, 72–77. 10.1016/j.epidem.2014.08.006 (2015). 25843388 10.1016/j.epidem.2014.08.006

[5] Vanhems, P. et al. Estimating potential infection transmission routes in hospital wards using wearable proximity sensors. *PLoS ONE* **8**, e73970. 10.1371/journal.pone.0073970 (2013). 24040129 10.1371/journal.pone.0073970 PMC3770639

[6] Génois, M. et al. Data on face-to-face contacts in an office building suggest a low-cost vaccination strategy based on community linkers. *Netw. Sci.* **3**, 326–347 (2015).

[7] Namtirtha, A., Dutta, A. & Dutta, B. Identifying influential spreaders in complex networks based on kshell hybrid method. *Phys. A* **499**, 310–324. 10.1016/j.physa.2018.02.016 (2018).

[8] Ullah, A. et al. Identification of nodes influence based on global structure model in complex networks. *Sci. Rep.* **11**, 1–11. 10.1038/s41598-021-84684-x (2021). 33731720 10.1038/s41598-021-84684-x PMC7969936

[9] Bae, J. & Kim, S. Identifying and ranking influential spreaders in complex networks by neighborhood coreness. *Phys. A* **395**, 549–559. 10.1016/j.physa.2013.10.047 (2014).

[10] Li, M. et al. Identifying and ranking influential spreaders in complex networks by combining a local-degree sum and the clustering coefficient. *Int. J. Mod. Phys. B* **32**, 1850118. 10.1142/S0217979218501187 (2018).

[11] Chen, D.-B., Gao, H., Lü, L. & Zhou, T. Identifying influential nodes in large-scale directed networks: the role of clustering. *PLoS ONE* **8**, e77455. 10.1371/journal.pone.0077455 (2013). 24204833 10.1371/journal.pone.0077455 PMC3814409

[12] Wang, X., Slamu, W., Guo, W., Wang, S. & Ren, Y. A novel semi local measure of identifying influential nodes in complex networks. *Chaos Solitons Fract.* **158**, 112037. 10.1016/j.chaos.2022.112037 (2022).

[13] Namtirtha, A., Dutta, B. & Dutta, A. Semi-global triangular centrality measure for identifying the influential spreaders from undirected complex networks. *Expert Syst. Appl.* **206**, 117791. 10.1016/j.eswa.2022.117791 (2022).

[14] Freeman, L. C. A set of measures of centrality based on betweenness. *Sociometry* **1977**, 35–41 (1977).

[15] Sabidussi, G. The centrality index of a graph. *Psychometrika* **31**, 581–603. 10.1007/BF02289527 (1966). 5232444 10.1007/BF02289527

[16] Kitsak, M. et al. Identification of influential spreaders in complex networks. *Nat. Phys.* **6**, 888–893. 10.1038/NPHYS1746 (2010).

[17] Ahajjam, S. & Badir, H. Identification of influential spreaders in complex networks using hybridrank algorithm. *Sci. Rep.* **8**, 1–10. 10.1038/s41598-018-30310-2 (2018). 30093716 10.1038/s41598-018-30310-2 PMC6085314

[18] Bonacich, P. & Lloyd, P. Eigenvector-like measures of centrality for asymmetric relations. *Soc. Netw.* **23**, 191–201. 10.1016/S0378-8733(01)00038-7 (2001).

[19] Shetty, R. D., Bhattacharjee, S., Dutta, A. & Namtirtha, A. Gsi: an influential node detection approach in heterogeneous network using covid-19 as use case. *IEEE Trans. Comput. Soc. Syst.*10.1109/TCSS.2022.3180177 (2022).

[20] Wei, B., Liu, J., Wei, D., Gao, C. & Deng, Y. Weighted k-shell decomposition for complex networks based on potential edge weights. *Phys. A* **420**, 277–283. 10.1016/j.physa.2014.11.012 (2015).

[21] Wang, J., Hou, X., Li, K. & Ding, Y. A novel weight neighborhood centrality algorithm for identifying influential spreaders in complex networks. *Phys. A* **475**, 88–105. 10.1016/j.physa.2017.02.007 (2017).

[22] Namtirtha, A., Dutta, A., Dutta, B., Sundararajan, A. & Simmhan, Y. Best influential spreaders identification using network global structural properties. *Sci. Rep.* **11**, 1–15. 10.1038/s41598-021-81614-9 (2021). 33500445 10.1038/s41598-021-81614-9 PMC7838212

[23] Namtirtha, A., Dutta, A. & Dutta, B. Weighted kshell degree neighborhood: a new method for identifying the influential spreaders from a variety of complex network connectivity structures. *Expert Syst. Appl.* **139**, 112859. 10.1016/j.eswa.2019.112859 (2020).

[24] Namtirtha, A., Dutta, A. & Dutta, B. Weighted kshell degree neighborhood method: an approach independent of completeness of global network structure for identifying the influential spreaders. In *2018 10th International Conference on Communication Systems & Networks (COMSNETS)* 81–88 (IEEE, 2018).

[25] Yoneki, E. & Crowcroft, J. Epimap: towards quantifying contact networks for understanding epidemiology in developing countries. *Ad Hoc Netw.* **13**, 83–93. 10.1016/j.adhoc.2012.06.003 (2014).

[26] Bansal, S., Read, J., Pourbohloul, B. & Meyers, L. A. The dynamic nature of contact networks in infectious disease epidemiology. *J. Biol. Dyn.* **4**, 478–489. 10.1080/17513758.2010.503376 (2010). 22877143 10.1080/17513758.2010.503376

[27] Danon, L. et al. Networks and the epidemiology of infectious disease. *Interdiscipl. Perspect. Infect. Dis.* **2011**, 78. 10.1155/2011/284909 (2011). 10.1155/2011/284909 PMC3062985 21437001

[28] Garas, A., Schweitzer, F. & Havlin, S. A k-shell decomposition method for weighted networks. *New J. Phys.* **14**, 083030. 10.1088/1367-2630/14/8/083030 (2012).

[29] Bollobás, B. The evolution of random graphs–the giant component. In *Random Graphs, vol. 184* 130–159 (Cambridge University Press, 2001).

[30] Meng, L., Xu, G., Yang, P. & Tu, D. A novel potential edge weight method for identifying influential nodes in complex networks based on neighborhood and position. *J. Comput. Sci.* **60**, 101591. 10.1016/j.jocs.2022.101591 (2022).

[31] Opsahl, T., Agneessens, F. & Skvoretz, J. Node centrality in weighted networks: generalizing degree and shortest paths. *Soc. Netw.* **32**, 245–251. 10.1016/j.socnet.2010.03.006 (2010).

[32] Barrat, A., Barthelemy, M., Vespignani, A. The architecture of complex weighted networks: Measurements and models. In *Large Scale Structure and Dynamics of Complex Networks: From information Technology to Finance and Natural Science* 67–92 (World Scientific, 2007).

[33] Freeman, L. C. Centrality in social networks conceptual clarification. *Soc. Netw.* **1**, 215–239 (1978).

[34] Lee, T., Lee, H.-R., Hwang, K. Identifying superspreaders for epidemics using r0-adjusted network centrality. In *2013 Winter Simulations Conference (WSC)* 2239–2249 (IEEE, 2013).

[35] Newman, M. E. Scientific collaboration networks. ii. Shortest paths, weighted networks, and centrality. *Phys. Rev. E* **64**, 016132 (2001). 10.1103/PhysRevE.64.016132 11461356

[36] Mirzasoleiman, B., Babaei, M., Jalili, M. & Safari, M. Cascaded failures in weighted networks. *Phys. Rev. E* **84**, 046114. 10.1103/PhysRevE.84.046114 (2011). 10.1103/PhysRevE.84.046114 22181234

[37] He, Z., Liu, S. & Zhan, M. Dynamical robustness analysis of weighted complex networks. *Phys. A* **392**, 4181–4191. 10.1016/j.physa.2013.05.005 (2013).

[38] Zhang, C.-J. & Zeng, A. Network skeleton for synchronization: identifying redundant connections. *Phys. A* **402**, 180–185. 10.1016/j.physa.2014.02.002 (2014).

[39] Stein, R. A. Super-spreaders in infectious diseases. *Int. J. Infect. Dis.* **15**, e510–e513. 10.1016/j.ijid.2010.06.020 (2011). 21737332 10.1016/j.ijid.2010.06.020 PMC7110524

[40] Pastor-Satorras, R., Castellano, C., Van Mieghem, P. & Vespignani, A. Epidemic processes in complex networks. *Rev. Mod. Phys.* **87**, 925. 10.1103/RevModPhys.87.925 (2015).

[41] Schimit, P. H. & Pereira, F. H. Disease spreading in complex networks: a numerical study with principal component analysis. *Expert Syst. Appl.* **97**, 41–50. 10.1016/j.eswa.2017.12.021 (2018). 32288338 10.1016/j.eswa.2017.12.021 PMC7126495

[42] Shetty, R.D., Bhattacharjee, S. A weighted hybrid centrality for identifying influential individuals in contact networks. In *2022 IEEE International Conference on Electronics, Computing and Communication Technologies (CONECCT)* 1–6. 10.1109/CONECCT55679.2022.9865749 (IEEE, 2022).

[43] Christley, R. M. et al. Infection in social networks: using network analysis to identify high-risk individuals. *Am. J. Epidemiol.* **162**, 1024–1031. 10.1093/aje/kwi308 (2005). 16177140 10.1093/aje/kwi308

[44] Allard, A., Serrano, M., García-Pérez, G. & Boguñá, M. The geometric nature of weights in real complex networks. *Nat. Commun.* **8**, 1–8. 10.1038/ncomms14103 (2017). 28098155 10.1038/ncomms14103 PMC5253659

[45] Brockmann, D. & Helbing, D. The hidden geometry of complex, network-driven contagion phenomena. *Science* **342**, 1337–1342 (2013). 24337289 10.1126/science.1245200

[46] Holme, P. & Saramäki, J. Temporal networks. *Phys. Rep.* **519**, 97–125. 10.1016/j.physrep.2012.03.001 (2012).

[47] Iannelli, F., Koher, A., Brockmann, D., Hövel, P. & Sokolov, I. M. Effective distances for epidemics spreading on complex networks. *Phys. Rev. E* **95**, 012313. 10.1103/PhysRevE.95.012313 (2017). 28208446 10.1103/PhysRevE.95.012313 PMC7217543

[48] Ullah, A. et al. A novel relevance-based information interaction model for community detection in complex networks. *Expert Syst. Appl.* **196**, 116607. 10.1016/j.eswa.2022.116607 (2022).

[49] Sociopatterns Datasets (2023, accessed 24 Jul 2023). http://www.sociopatterns.org/datasets/

[50] Ciaperoni, M. et al. Relevance of temporal cores for epidemic spread in temporal networks. *Sci. Rep.* **10**, 1–15. 10.1038/s41598-020-69464-3 (2020). 32719352 10.1038/s41598-020-69464-3 PMC7385111

[51] Colosi, E. et al. Screening and vaccination against covid-19 to minimise school closure: a modelling study. *Lancet. Infect. Dis*10.1016/S1473-3099(22)00138-4 (2022). 35378075 10.1016/S1473-3099(22)00138-4 PMC8975262

[52] Shetty, R., Bhattacharjee, S. Weighted network analysis example code (2025, accessed 10 Jan 2025). https://github.com/RamyaDShetty/Weighted-Network-Analysis.

[53] Cattuto, C. et al. Dynamics of person-to-person interactions from distributed rfid sensor networks. *PLoS ONE* **5**, e11596. 10.1371/journal.pone.0011596 (2010). 20657651 10.1371/journal.pone.0011596 PMC2904704

[54] G’enois, M. & Barrat, A. Can co-location be used as a proxy for face-to-face contacts?. *EPJ Data Sci.* **7**, 11. 10.1140/epjds/s13688-018-0140-1 (2018).

[55] Genois, M. et al. Data on face-to-face contacts in an office building suggest a low-cost vaccination strategy based on community linkers. *Netw. Sci.* **3**, 326–347. 10.1017/nws.2015.10 (2015).

[56] StehlÃ, J. et al. High-resolution measurements of face-to-face contact patterns in a primary school. *PLOS ONE* **6**, e23176. 10.1371/journal.pone.0023176 (2011). 21858018 10.1371/journal.pone.0023176 PMC3156713

[57] Fournet, J. & Barrat, A. Contact patterns among high school students. *PLoS ONE* **9**, e107878. 10.1371/journal.pone.0107878 (2014). 25226026 10.1371/journal.pone.0107878 PMC4167238

[58] Mastrandrea, R., Fournet, J. & Barrat, A. Contact patterns in a high school: a comparison between data collected using wearable sensors, contact diaries and friendship surveys. *PLoS ONE* **10**, e0136497. 10.1371/journal.pone.0136497 (2015). 26325289 10.1371/journal.pone.0136497 PMC4556655

[59] Moreno, Y., Pastor-Satorras, R. & Vespignani, A. Epidemic outbreaks in complex heterogeneous networks. *Eur. Phys. J. B-Condens. Matter Compl. Syst.* **26**, 521–529. 10.1140/epjb/e20020122 (2002).

[60] Kendall, M. G. The treatment of ties in ranking problems. *Biometrika* **33**, 239–251 (1945). 21006841 10.1093/biomet/33.3.239

[61] Liu, Y., Tang, M., Zhou, T. & Do, Y. Identify influential spreaders in complex networks, the role of neighborhood. *Phys. A* **452**, 289–298. 10.1016/j.physa.2016.02.028 (2016).

